# Acrolein Induces Systemic Coagulopathy *via* Autophagy-dependent Secretion of von Willebrand Factor in Mice after Traumatic Brain Injury

**DOI:** 10.1007/s12264-021-00681-0

**Published:** 2021-05-03

**Authors:** Wenxing Cui, Xun Wu, Dayun Feng, Jianing Luo, Yingwu Shi, Wei Guo, Haixiao Liu, Qiang Wang, Liang Wang, Shunnan Ge, Yan Qu

**Affiliations:** Department of Neurosurgery, Tangdu Hospital, Fourth Military Medical University, Xi’an, 710038 China

**Keywords:** Traumatic brain injury, Coagulopathy, Autophagy, Acrolein, Von Willebrand factor

## Abstract

Traumatic brain injury (TBI)-induced coagulopathy has increasingly been recognized as a significant risk factor for poor outcomes, but the pathogenesis remains poorly understood. In this study, we aimed to investigate the causal role of acrolein, a typical lipid peroxidation product, in TBI-induced coagulopathy, and further explore the underlying molecular mechanisms. We found that the level of plasma acrolein in TBI patients suffering from coagulopathy was higher than that in those without coagulopathy. Using a controlled cortical impact mouse model, we demonstrated that the acrolein scavenger phenelzine prevented TBI-induced coagulopathy and recombinant ADAMTS-13 prevented acrolein-induced coagulopathy by cleaving von Willebrand factor (VWF). Our results showed that acrolein may contribute to an early hypercoagulable state after TBI by regulating VWF secretion. mRNA sequencing (mRNA-seq) and transcriptome analysis indicated that acrolein over-activated autophagy, and subsequent experiments revealed that acrolein activated autophagy partly by regulating the Akt/mTOR pathway. In addition, we demonstrated that acrolein was produced in the perilesional cortex, affected endothelial cell integrity, and disrupted the blood-brain barrier. In conclusion, in this study we uncovered a novel pro-coagulant effect of acrolein that may contribute to TBI-induced coagulopathy and vascular leakage, providing an alternative therapeutic target.

**Supplementary Information:**

The online version contains supplementary material available at 10.1007/s12264-021-00681-0.

## Introduction

Traumatic brain injury (TBI) constitutes a significant proportion of global injuries, and remains one of the major causes of traumatic death and disability [1]. Despite advances in the treatment of TBI, a high risk of poor outcomes still exists in these patients. There are areas regarding our comprehension of the pathogenesis and optimal treatment strategies of TBI that need improvement. Coagulopathy is a common secondary injury in TBI patients, occurring in 33% to 66% of cases, due to the different detection methods and definitions [2, 3]. TBI-induced coagulopathy is consistently associated with poor outcomes [4, 5], and these patients tend to suffer from progressive intracranial hemorrhage and microvascular thrombosis [6, 7].

TBI-induced coagulopathy follows a distinct pathogenic pathway. First, the incidence of coagulopathy after TBI is higher than that after traumatic injuries of other organs. Second, isolated TBI can also induce early and systemic coagulopathy, even without substantial blood loss and hemodilution because of fluid resuscitation [8]. Previous studies have shown that TBI-induced coagulopathy manifests as a hypercoagulable state induced by pro-coagulant molecules (such as tissue factors, phosphatidylserine, and cardiolipin) released from injured brain tissue; this then develops into a late consumptive hypocoagulable state [5, 8–10]. However, the removal of these pro-coagulant molecules does not completely correct the coagulation dysfunction [11], which suggests that there may be other molecules involved in TBI-induced coagulopathy.

It is widely known that the brain is the most lipid-rich organ (approximately 50% of the brain dry weight) [12]. Oxidative stress is a crucial contributor to secondary insult following TBI [13], and the role of lipid peroxidation in the course of this pathology is gradually being recognized [14]. However, whether lipid peroxidation products affect coagulation in the early stage of TBI is unclear. Acrolein, a highly active unsaturated aldehyde [15], is abundantly produced during the acute phase of TBI [16], and the strong covalent bonds to cellular and mitochondrial proteins can impair the structural and functional integrity in cells [17–19]. Other evidence also shows that acrolein is closely associated with the occurrence of thrombotic diseases [20], which indicates its role in stimulation of the coagulation cascade. Here, we hypothesized that acrolein promotes TBI-induced coagulopathy in the early stage. In the current study, we first identified a potential relationship between plasma acrolein levels in TBI patients and coagulopathy. Next, *in vitro* and *in vivo* studies were performed to explore the novel role of acrolein in contributing to secondary coagulopathy after TBI in mice. Then, we performed transcriptome sequencing to explore the possible mechanism of acrolein-induced coagulopathy and validated the findings. Finally, we verified that phenelzine, an acrolein scavenger, has great therapeutic potential.

## Materials and Methods

### Animals and Ethics

All experimental procedures strictly followed the guidelines of the National Institutes of Health Guide for the Care and Use of Laboratory Animals and were approved by the Ethics Committee of the Fourth Military Medical University. Eight- to twelve-week-old healthy adult male C57BL/6J mice weighing 20 g–25 g (wild type) were purchased from the Animal Center of the Fourth Military Medical University. All mice were kept at a constant humidity (60%), temperature (18°C–22°C), with a regular 12-h light/dark cycle and free access to food and water in a specific pathogen-free animal room. This study was approved by the Ethics Committee of Tangdu Hospital, Fourth Military Medical University (201907-03), and was registered on clinicaltrials.gov (NCT04274777).

### TBI Procedure and Drug Administration

A controlled cortical impact (CCI) model for TBI was established as previously described [21]. Briefly, each mouse was anesthetized with 2% pentobarbital sodium, and underwent TBI surgery with the CCI device (68099 Precision Strike, RWD, Shenzhen, Guangzhou, China). Each mouse was fixed on a stereotactic device, and the skull was exposed. A bone window 2 mm in diameter was drilled with a grinder at 1.5 mm behind the bregma and 1.5 mm on the right side, while the integrity of the dura was maintained. The round metal tip perpendicularly struck the exposed cortical surface at a velocity of 3 m/s and remained for 0.2 s, leading to a depth of 1.5 mm. Then, tissue adhesive was used to cover the damaged cortex. Mice in the sham injury underwent the same procedure without the use of the CCI device. Phenelzine (MedChemExpress, HY-B1018A) was dissolved in 0.9% saline. Mice in the phenelzine treatment group were intraperitoneally injected with 10 mg/kg phenelzine immediately after TBI [22]. Mice in the recombinant human ADAMTS-13 (rhADAMTS-13, R&D, 4245-AD-020) treatment group were intraperitoneally injected with 200 μg/Kg rhADAMTS-13 immediately after TBI [23]. Mice in the positive control group were injected with acrolein through the tail vein [24], while mice in the vehicle group were injected with saline through the tail vein.

### Western Blot Analysis

Western blot analysis was performed as previously described [25]. The selected tissue samples were homogenized in lysis buffer containing 1% protease inhibitor. Protein concentrations were measured using a BCA protein assay kit (Thermo Scientific; UA276918). Proteins were separated on SDS-PAGE gels, and transferred onto PVDF membranes (Millipore, Billerica, MA), which were then incubated with primary antibodies at 4°C overnight. After 3 × 5-min washes in TBST, the membranes were probed with the appropriate horseradish peroxidase-conjugated anti-rabbit or anti-mouse secondary antibodies (1:5000, 27°C, 2 h). Protein signals were exposed with a BioRad imaging system (Bio-Rad, Hercules, CA). Images were analyzed using ImageJ. The primary antibodies used were as follows: anti-acrolein (1:1000, ab240918, Abcam), anti-Atg5 (1:1000, 12994S, Cell Signaling), anti-Atg7 (1:1000, 8558S, Cell Signaling), anti-Beclin 1 (1:1000, 3738S, Cell Signaling), anti-LC3 (1:1000,2775S, Cell Signaling), anti-Akt (1:1000, 4691S, Cell Signaling), anti-p Akt (1:1000, 4060S, Cell Signaling), anti-mTOR (1:1000, 2983S, Cell Signaling), anti-p mTOR (1:1000,2971S, Cell Signaling), anti-Occludin (1:1000, 27260-1-AP, Proteintech), anti-ZO-1 (1:200, ab96587, Abcam), and anti-β-actin (1:3000, wh096194, Wanleibio).

### ELISA

The levels of acrolein in the plasma of TBI patients and mice were measured using ELISA kits (MBS7213206, Biocompare; JL47824, Jianglai, respectively). The levels of VWF and D-dimer in mouse plasma were determined using an Elisa kit (E-EL-M1247c, Elabscience; JL20160, Jianglai, respectively). Whole blood from patients or mice was centrifuged at 1500 g for 15 min to obtain plasma, and then stored at – 80°C. The levels of VWF in human endothelial cell medium were determined using a VWF ELISA kit (ab108918, Abcam). The ELISA procedures were carried out in strict accordance with the instructions.

### Immunofluorescence Staining

Immunofluorescence staining was performed as previously described [25]. Briefly, mice were sacrificed 24 h after TBI and perfused with 4% paraformaldehyde. The brain was removed and fixed with 4% paraformaldehyde at 4°C overnight, and then dehydrated in 10%, 20%, and 30% sucrose. Next, the brain was cut into 15–25 μm sections, and incubated in 0.1% Triton X-100 for 30 min, followed by incubation in 10% donkey serum for 2 h. The sections were incubated with the mouse anti-acrolein (1:200, ab48501, Abcam), anti-glial fibrillary acidic protein (GFAP; 1:200; Invitrogen, USA), and anti-CD31 (1:200, ab222783, Abcam) at 4°C overnight. All sections were analyzed under a fluorescence microscope (A1 Si, Nikon) in a blinded manner. Representative images were from three independent experiments using six mice.

### Clotting Time

Careful collection of blood is vital for analyzing coagulation. We collected blood as previously described [26]. Briefly, the mice were anesthetized using 2% pentobarbital sodium. Then, they were placed in dorsal recumbency, and the thorax exposed by cutting the skin around the rib cage. Next, we carefully and quickly inserted a needle into the right ventricle to draw the blood. Immediately, we transferred the blood from the syringe into the Century Clot blood coagulation and platelet function analyzer (Shijiyikang, Tianjin). This device uses a sensitive mechanical sensing system to detect viscoelasticity, and continuously monitors the coagulation process of blood samples *in vitro* in real time, allowing the accurate determination of clotting time.

### Quantitative Real-time PCR (qRT-PCR)

qRT-PCR was performed as previously described [27]. Total RNA was isolated from cells using TRIzol reagent (Invitrogen). Reverse transcription was conducted to obtain cDNAs using HiScript II Q RT SuperMix for qRT-PCR (+gDNA wiper) (Vazyme, USA). qRT-PCR was conducted using an iQTM 5 Optical Module Real-Time PCR Detection System (Bio-Rad, USA). Gene expression was normalized to β-actin and calculated using the 2^-ΔΔCt^ method. The primer sequences are listed in Table S1.

### Cell Culture and CCK8 Cell Viability Assay

Human umbilical vein endothelial cells (HUVECs) were cultured in endothelial cell medium (1001, Science Cell), containing endothelial cell growth supplement (Cat. No. 1052), in a humidified incubator with 5% CO_2_ at 37°C. The CCK8 cell viability assay was performed as previously described [28]. After exposing the cells to acrolein for 6 h, CCK8 reagent was added to each well. After incubation for 2 h at 37°C, we measured the absorbance at 450 nm using a plate reader. The survival rate of the untreated cells was set at 100%.

### Transwell Assay

A transwell assay was used to measure endothelial permeability as previously described [23]. HUVECs were seeded onto inserts coated with collagen. After reaching confluence, the cells were incubated with a concentration gradient of acrolein (25, 50, and 100 μmol/L) at 37°C for 6 h. Next, the cells were incubated with 1 mg/mL FITC-dextran at 37°C for 1 h. Then, the insert was removed, and 100 µL of liquid in the bottom receiving plate was collected to measure the fluorescence intensity in a plate reader.

### Quantification of Lesion Volume and Edema

The lesion volume was measured as previously described [29]. Briefly, 24 h after TBI, the brains were collected and sections were prepared as above. Then, the sections were stained with Cresyl violet (for Nissl bodies). The lesion area was measured using ImageJ software.

Brain water content was quantified by the wet/dry method one day after TBI and calculated as a percentage using the following equation: brain water content = (wet weight − dry weight)/wet weight × 100% [29].

### Evans Blue Extravasation Analysis

Evans blue extravasation was used to assess blood-brain barrier (BBB) integrity as previously described [30]. Briefly, at 24 h post-TBI, Evans blue was intravenously injected and allowed to circulate for 1 h. Then, the mice were sacrificed and perfused with PBS. Afterward, the brains were isolated and homogenized with trichloroacetic acid solution. Next, the homogenates were centrifuged at 12,000×g for 30 min, and the OD of the supernatant was measured at 610 nm using a microplate reader.

### Modified Neurological Severity Score

The modified neurological severity score (mNSS) was used to assess neurological functional impairment as previously described [31]. The mNSS, including motor, sensory, reflex, and balance tests, ranged from 0 to 18 (0: normal score; 18: maximal deficit score). The scoring was conducted at 6 h and 1, 3, 5, and 7 days after TBI by two observers who were blinded to the groups.

### Patients and Ethical Considerations

This prospective study was conducted from September 2019 to March 2020 and approved by the Ethics Committee of Tangdu Hospital, Fourth Military Medical University, and was registered on clinicaltrials.gov. Two milliliters of venous blood were collected from TBI patients after admission. The clinical data of the patients were collected and included age, gender, admission Glasgow Coma Scale, pupil reaction at admission, medical history, and biochemical tests [activated partial thromboplastin time (aPTT), international normalized ratio (INR), platelet count, RBC, HCT, GLU, AST, and ALT]. Traumatic coagulopathy was defined as aPTT >36 s and/or INR >1.2 and/or platelet count <100 × 10^9^ per liter, based on a previous study [32]. The main inclusion criteria were patients with mild, moderate, or severe TBI. The exclusion criteria were as follows: (1) age <16 years or >80 years; (2) interval from injury to admission >24 h; (3) severe systemic diseases including uremia, cirrhosis, and malignant tumors; (4) ischemic or hemorrhagic vascular disease occurring within half a year; and (5) a medical history of taking anticoagulants or antiplatelet drugs and a history of smoking.

### Statistical Analysis

Statistical analysis was performed using IBM SPSS Statistics 20.0 software (IBM, New York, NY). Continuous variables are presented as the mean ± SEM. Categorical data are presented as the frequency (percentages). Univariate analysis was performed to find significant variables, which were entered into the multivariate logistic regression to identify the independent risk factors for coagulopathy. The levels of plasma acrolein were classified by applying ROC curve analysis based on coagulopathy. Two independent groups were compared using unpaired two-tailed Student’s *t* test, while multiple groups were analyzed using one-way analysis of variance, followed by the Tukey *post hoc* test. Neurobehavioral data were analyzed using the Kruskal–Wallis one-way analysis of variance on ranks followed by the Student–Newman–Keuls test. A value of *P* < 0.05 was defined as statistically significant.

## Results

### The Plasma Acrolein Level in TBI Patients is Correlated with Coagulopathy

**Fig. 1:**
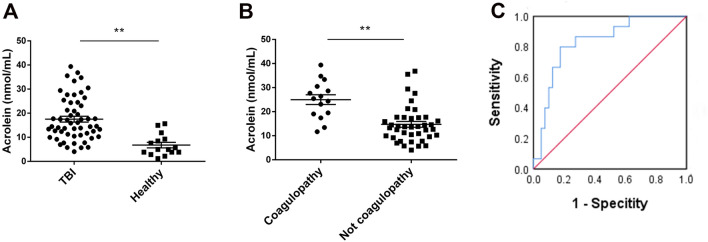
Plasma acrolein levels in TBI patients and their ability to discriminate coagulopathy. **A** Plasma acrolein concentrations in healthy controls and TBI patients (*n* = 60, **P < 0.01). **B** Plasma acrolein concentrations in TBI patients suffering from coagulopathy at admission and those without coagulopathy (*n* = 55, **P < 0.01). **C** Analysis of the discriminative ability of plasma acrolein concentrations for patients at risk of coagulopathy after TBI using the receiver operating characteristic curve.

A total of 55 TBI patients were enrolled in this prospective clinical study. First, we found that the plasma acrolein level in TBI patients was higher than that in normal individuals (*P* < 0.01; Fig. 1A). Further, plasma acrolein levels were higher in patients with coagulopathy than those without coagulopathy (*P* < 0.01; Fig. 1B). ROC curve analysis was used to test the discriminative ability of plasma acrolein levels for coagulopathy (Fig. 1C). An optimal cutoff value of acrolein concentration (18.4 nmol/mL) was chosen, which discriminated TBI patients at risk of coagulopathy with 80.0% sensitivity and 82.5% specificity with an area under the curve of 0.830. According to the univariate analysis, older age (*P* = 0.005), reduced Glasgow coma score (*P* = 0.047), abnormal pupil reaction (*P* = 0.037), abnormal RBC (*P* = 0.014), abnormal HCT (*P* = 0.008), abnormal AST (*P* = 0.013), abnormal ALT (*P* = 0.004), and increased plasma acrolein level (*P* < 0.001) were associated with coagulopathy (Table 1). An increased plasma acrolein level (OR = 1.260, 95% CI 1.098–1.447, *P* = 0.001; Table 1) remained an independent risk factor for coagulopathy. These clinical results strongly suggested that there is a positive correlation between the plasma acrolein level and TBI-induced coagulopathy.

**Table 1:** Characteristics of the study population.

	Study population (*n* = 55)	Coagulopathy (*n* = 15)	No coagulopathy (*n* = 40)	*P* value	Adjusted *P* value	Adjusted OR (95% CI)
Demographics						
Age, years (SD)	54.5 (12.0)	61.5 (9.5)	51.9 (11.8)	0.005	0.023	1.151 (1.019, 1.299)
Male, *n* (%)	40 (72.7)	12 (80.0)	28 (70.0)	0.458		
GCS (SD)	10.0 (3.5)	8.3 (3.8)	10.6 (3.2)	0.047		
Abnormal pupil reaction, *n* (%)	24 (43.6)	10 (66.7)	14 (35.0)	0.037		
Medical history						
Hypertension, *n* (%)	9 (16.4)	2 (13.3)	7 (17.5)	0.71		
Diabetes, *n* (%)	1 (1.8)	1 (6.7)	0 (0)	0.537		
Coronary artery disease, *n* (%)	2 (3.6)	1 (6.7)	1 (2.5)	0.462		
Mechanism of injury						
Motor vehicle, *n* (%)	26 (47.3)	9 (60.0)	17 (42.5)	0.247		
Fall, *n* (%)	27 (49.1)	6 (40.0)	21 (52.5)	0.409		
Assault, *n* (%)	1 (1.8)	0 (0)	1 (2.5)	0.537		
Laboratory biochemical examinations						
Abnormal RBC, *n* (%)	13 (23.6)	7 (46.7%)	6 (15.0)	0.014		
Abnormal HCT, *n* (%)	15 (27.3)	8 (53.3)	7 (17.5)	0.008	0.024	11.304 (1.380, 92.581)
Abnormal GLU, *n* (%)	14 (25.5)	6 (40.0)	8 (20.0)	0.129		
Abnormal AST, *n* (%)	29 (52.7)	12 (80.0)	17 (42.5)	0.013		
Abnormal ALT, *n* (%)	17 (30.9)	9 (60.0)	8 (20.0)	0.004		
Acrolein (ng/mL) (SD)	17.5 (8.9)	25.0 (7.9)	14.7 (7.6)	< 0.001	0.001	1.260 (1.098, 1.447)

### Upregulation of Acrolein in Peripheral Blood After TBI in Mice and the Association with an Early Hypercoagulable State

**Fig. 2:**
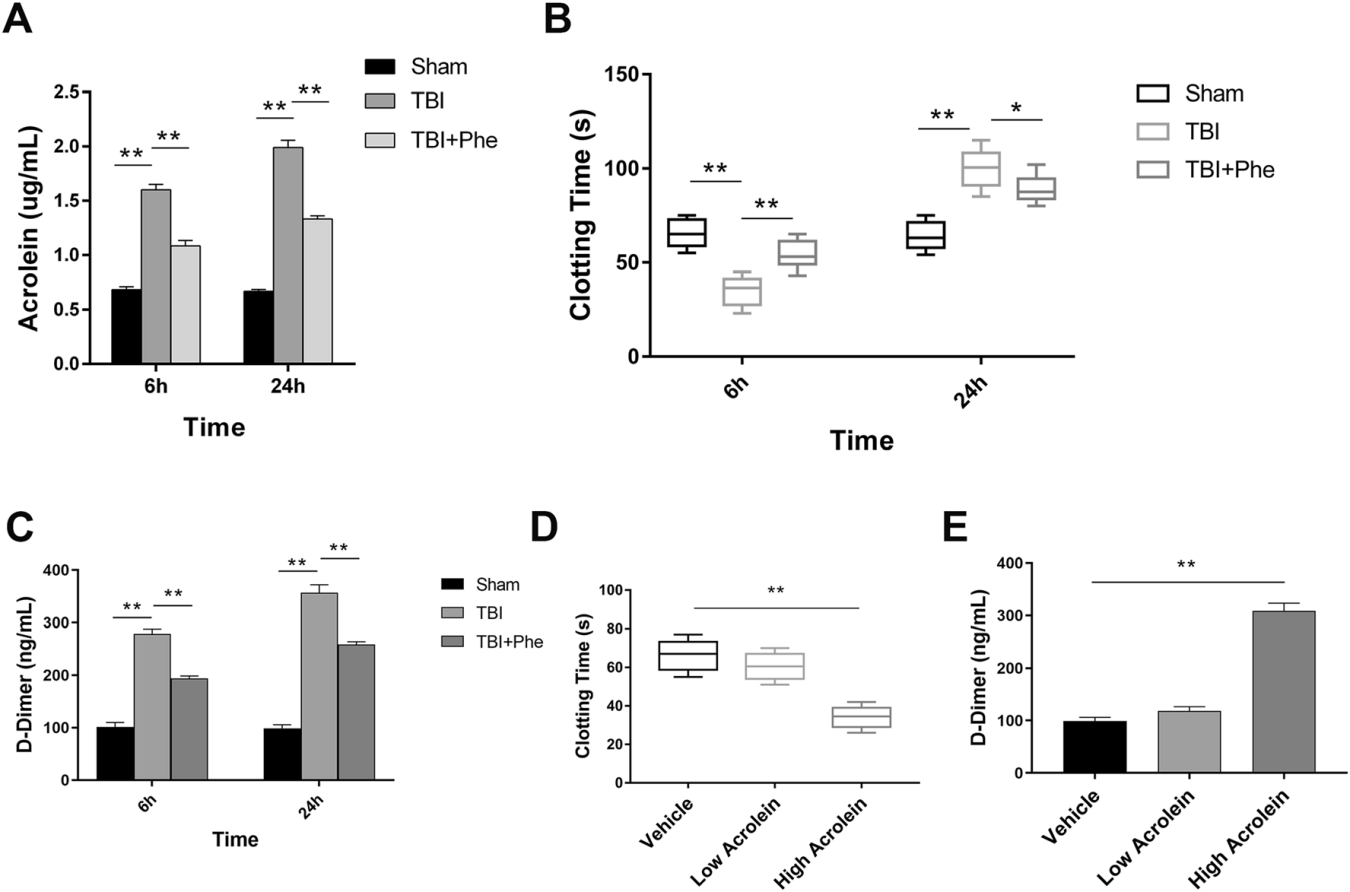
Upregulation of acrolein in peripheral blood after TBI in mice contributes to an early hypercoagulable state. **A** ELISA results showing the acrolein levels in peripheral blood at 6 h and 24 h in each group (Sham; TBI; TBI+Phe). **B** Clotting time at 6 h and 24 h in each group. **C** Plasma levels of D-dimer at 6 h in each group. **D** Clotting time at 6 h in each group (vehicle; low acrolein: 3 nmol; high acrolein: 30 nmol). **E** Plasma levels of D-dimer at 6 h in each group. Values are presented as the mean ± SEM, *n* = 6 per group, **P < 0.01.

First, ELISA was used to assess the alterations in acrolein with time after TBI with or without the administration of the acrolein scavenger phenelzine. The acrolein level in peripheral blood increased significantly at 6 h, and was cleared by phenelzine (Fig. 2A). To explore the effects of increasing acrolein on the clotting system, we selected two representative time points (6 h and 24 h) to measure the commonly used indicators of coagulation including clotting time and d-dimers, based on previous studies [11]. In mice, we found that the clotting time was shortened in the early stage (6 h) of trauma and prolonged in the later stage (24 h) (Fig. 2B). These changes were partly reversed by the administration of phenelzine after TBI (Fig. 2B). The level of plasma D-dimer increased significantly after TBI, and this abnormal elevation was mitigated by treatment with phenelzine (Fig. 2C). To further validate the role of acrolein in coagulation, uninjured mice were injected through the tail vein with different doses of acrolein (low dose: 3 nmol; high dose: 30 nmol). The selection of doses was based on the level of acrolein in peripheral blood after TBI, as measured by ELISA. Low-dose acrolein (3 nmol) and saline had no significant effect on clotting time or the level of plasma D-dimer 6 h after injection, while high-dose acrolein (30 nmol) significantly shortened the clotting time and increased the level of D-dimer (Fig. 2D, E). These results revealed that acrolein, a pro-coagulant, is produced at an early stage after TBI and induces an early hypercoagulable state, which in turn causes a consumptive hypo-coagulable state.

### Acrolein Induces Coagulopathy Partly by Regulating VWF Secretion

**Fig. 3:**
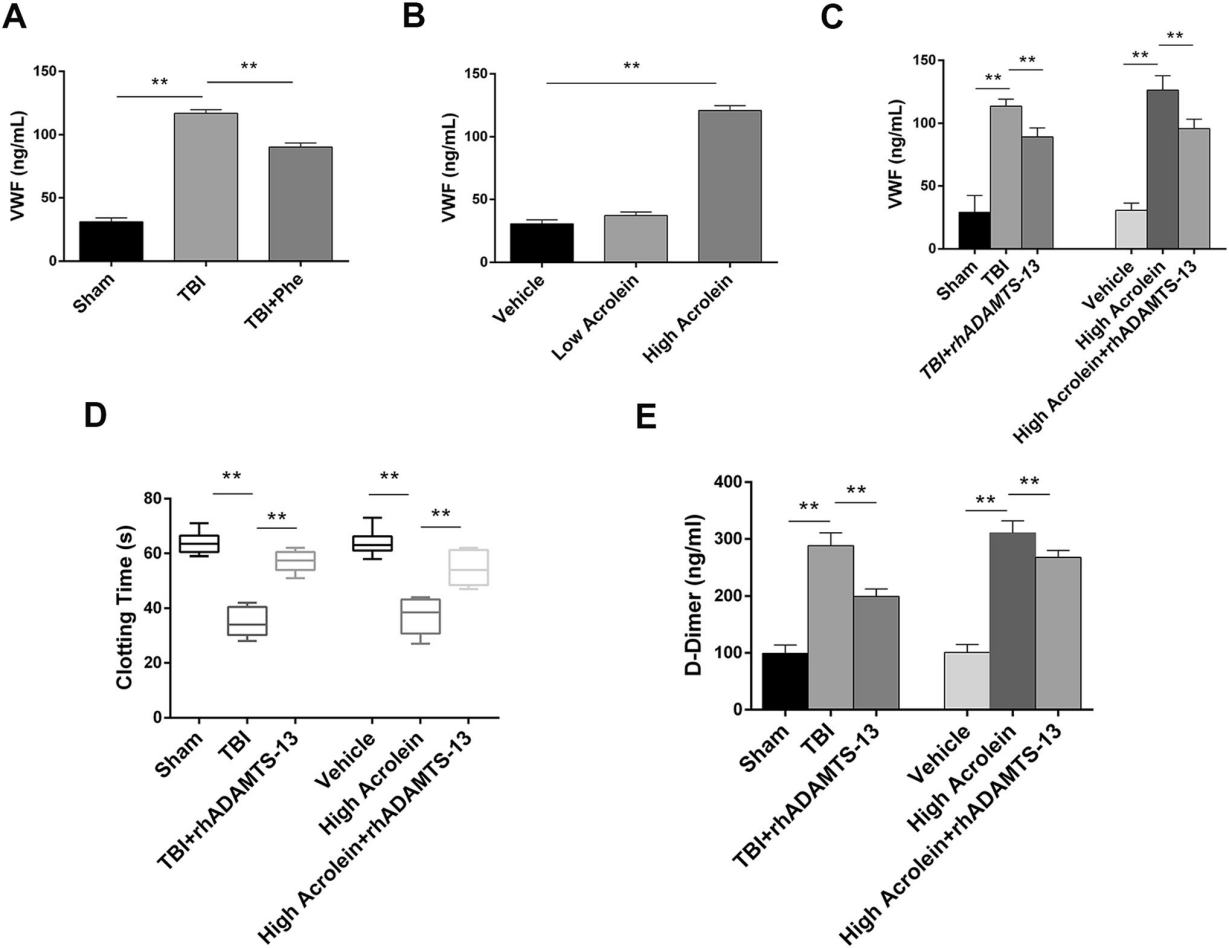
Acrolein induces coagulopathy partly by regulating VWF secretion. **A** VWF levels in mice treated with or without phenelzine after TBI and in sham mice. **B** VWF levels in mice infused with acrolein or PBS. **C** VWF levels in mice treated with rhADAMTS-13 after TBI or acrolein infusion. **D** Clotting times in the above treatment groups. **E** Plasma levels of D-dimer from mice in the above treatment groups. Values are presented as the mean ± SEM, *n* = 6 per group, **P < 0.01.

VWF is an adhesion molecule released and stored by endothelial cells, and is regarded as a marker of endothelial cell activation [33]. VWF is considered to be involved in coagulopathy and thromboembolic disease associated with trauma [23, 34, 35]. Therefore, we hypothesized that TBI-induced high-level plasma acrolein promotes coagulopathy *via* the release of VWF. First, the levels of circulating VWF were significantly increased at 6 h post-TBI, and this was partly reversed by phenelzine (Fig. 3A). Low-dose acrolein had no significant effects on circulating VWF levels, while high-dose acrolein significantly increased them (Fig. 3B). Next, the administration of rhADAMTS-13, an enzyme that cleaves VWF [36], significantly reduced circulating VWF levels after TBI. Similarly, rhADAMTS-13 reduced the increase in VWF induced by acrolein (Fig. 3C). Then, we explored whether rhADAMTS-13 reversed the coagulopathy induced by TBI or acrolein. We also found that rhADAMTS-13 partly reversed the abnormal clotting time and D-dimer level at 6 h post-TBI or acrolein injection (Fig. 3D, E). These results suggest that acrolein causes TBI-induced coagulopathy partly by promoting VWF secretion.

### Acrolein Activates the Autophagy Pathway in HUVECs as shown by mRNA-Seq Analysis

**Fig. 4:**
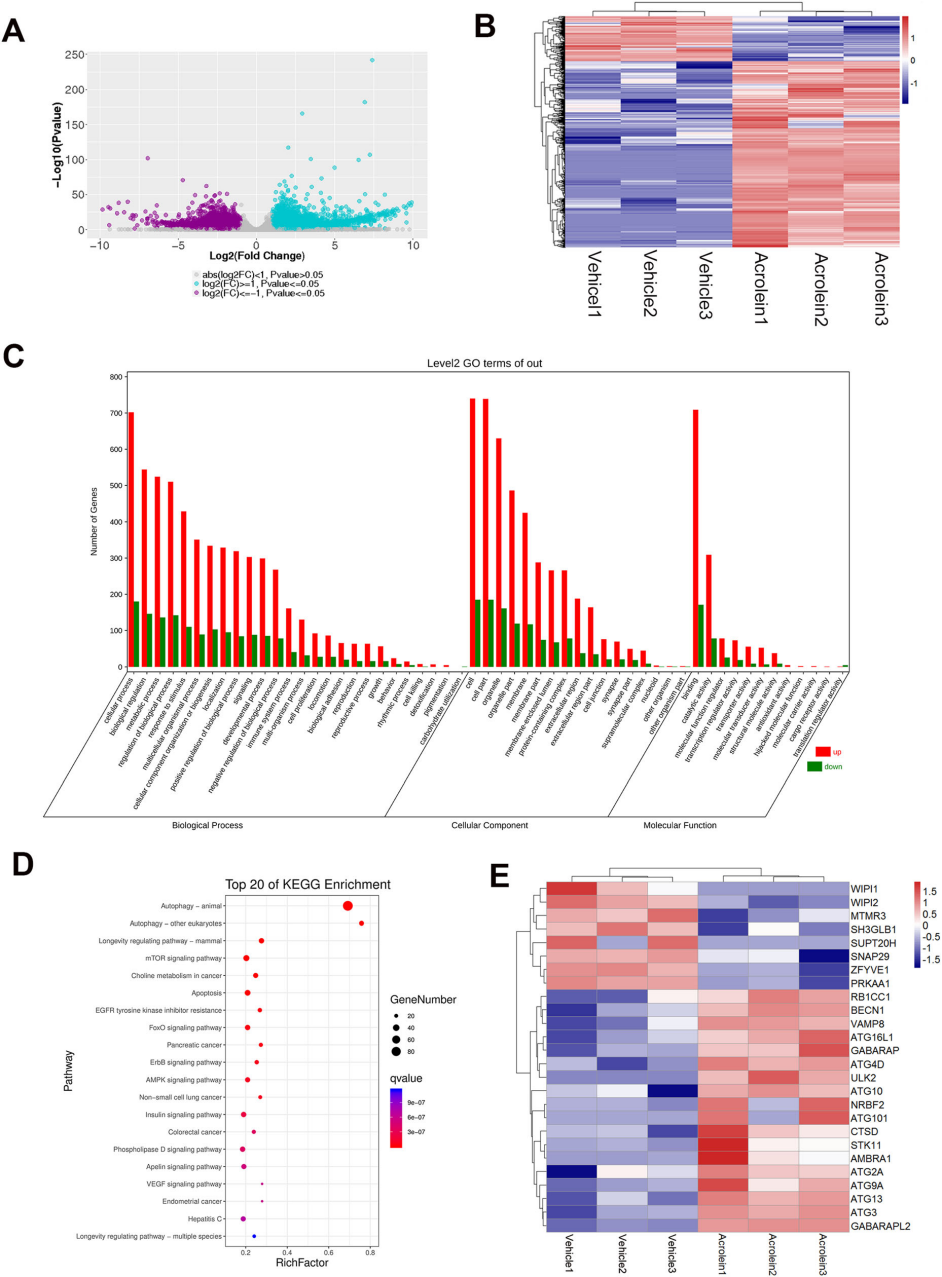
mRNA-Seq analysis showing that acrolein activates the autophagy pathway in HUVECs. **A, B** Volcano plot (**A**) and heatmap (**B**) of the control and acrolein treatment groups showing the total up-regulated and down-regulated genes. **C** GO terms for biological process, cellular components, and molecular function. **D** Top 20 from KEGG Enrichment. **E** Heat map showing autophagy-related gene expression.

To elucidate the effect of acrolein on HUVECs and VWF secretion, we first examined the toxicity of acrolein by CCK8 assays and found that the LD_50_ for acrolein was approximately 50 μmol/L after 6 h of treatment (Fig. S1A). Next, to investigate how acrolein activates HUVECs, we determined the profiles of transcriptomics changes in the control and acrolein treatment groups using RNA-seq analysis. Volcano plots and heatmaps of the two groups show the total upregulated and downregulated genes (Fig. 4A, B). We used GO (Gene Ontology) terms for biological process, cellular components, and molecular function to determine the functional enrichment of the differentially-expressed genes (Fig. 4C). Autophagy was most enriched in biological process and cellular components, and enzyme binding was most enriched in molecular function (Fig. S1B–D). Kyoto Encyclopedia of Genes Genomes (KEGG) enrichment analysis identified that autophagy was the most enriched pathway after acrolein treatment (Fig. 4D). Then, a heat map showing autophagy-related gene expression was generated to analyze the expression changes of each gene (Fig. 4E), and the trends in these typical genes was verified by qPCR (Fig. S4E). The mRNA-Seq analysis suggested that acrolein significantly activates the autophagy pathway.

### Acrolein Promotes the Release of VWF by Activating Autophagy

**Fig. 5:**
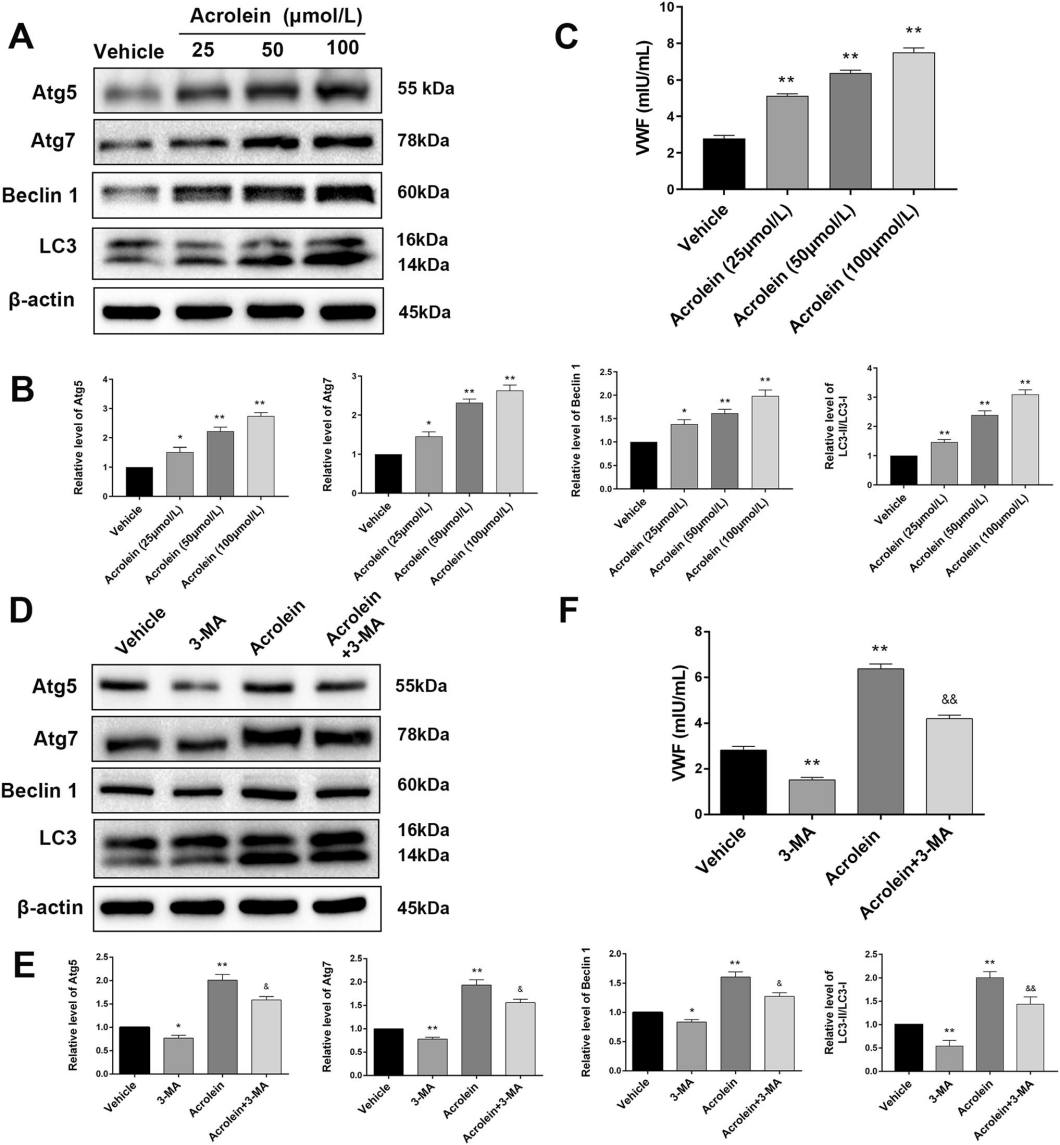
Acrolein regulates the secretion of VWF by activating autophagy. **A** Western blots of Atg5, Atg7, Becline1, and LC3 in HUVECs treated with different concentrations of acrolein (25, 50, and 100 μmol/L) and vehicle for 6 h. **B** Relative levels of these proteins expressed as percentages of β-actin. **C** Effects of acrolein on VWF secretion in the above groups. **D** Western blots of Atg5, Atg7, Beclin-1, and LC3 in HUVECs pretreated with or without 5 mmol/L 3-MA for 2 h, followed by treatment with acrolein (50 μmol/L) for 6 h. **E** Relative levels of these proteins expressed as percentages of β-actin. **F** Effects of 3-MA (5 mmol/L) on acrolein-induced VWF secretion in the above groups. Values are presented as the mean ± SEM, *n* = 3 per group, *P < 0.1, **P < 0.01.

Previous studies have shown that autophagy regulates endothelial VWF secretion [37], so we hypothesized that acrolein promotes the release of VWF by activating autophagy, thereby leading to coagulopathy. HUVECs were treated with different concentrations of acrolein (0, 25, 50, and 100 μmol/L) for 6 h. First, Western blotting was used to assess changes in the levels of autophagy-related molecules. The results revealed that acrolein upregulated the levels of Atg5, Atg7, and Beclin-1 and promoted the conversion of LC3-I to LC3-II in a dose-dependent manner (Fig. 5A, B). Using an ELISA-based approach on the culture medium, we found a significant increase in VWF secretion after treatment with acrolein, and this was also dose-dependent (Fig. 5C). Incubation of HUVECs with 3- Methyladenine (5 mmol/L) inhibited acrolein-induced autophagy (Fig. 5D, E), and acrolein-stimulated VWF secretion (Fig. 5F). The above results revealed that acrolein induces the release of VWF by activating autophagy.

### Acrolein Activates Autophagy *via* the Akt/mTOR Pathway

**Fig. 6:**
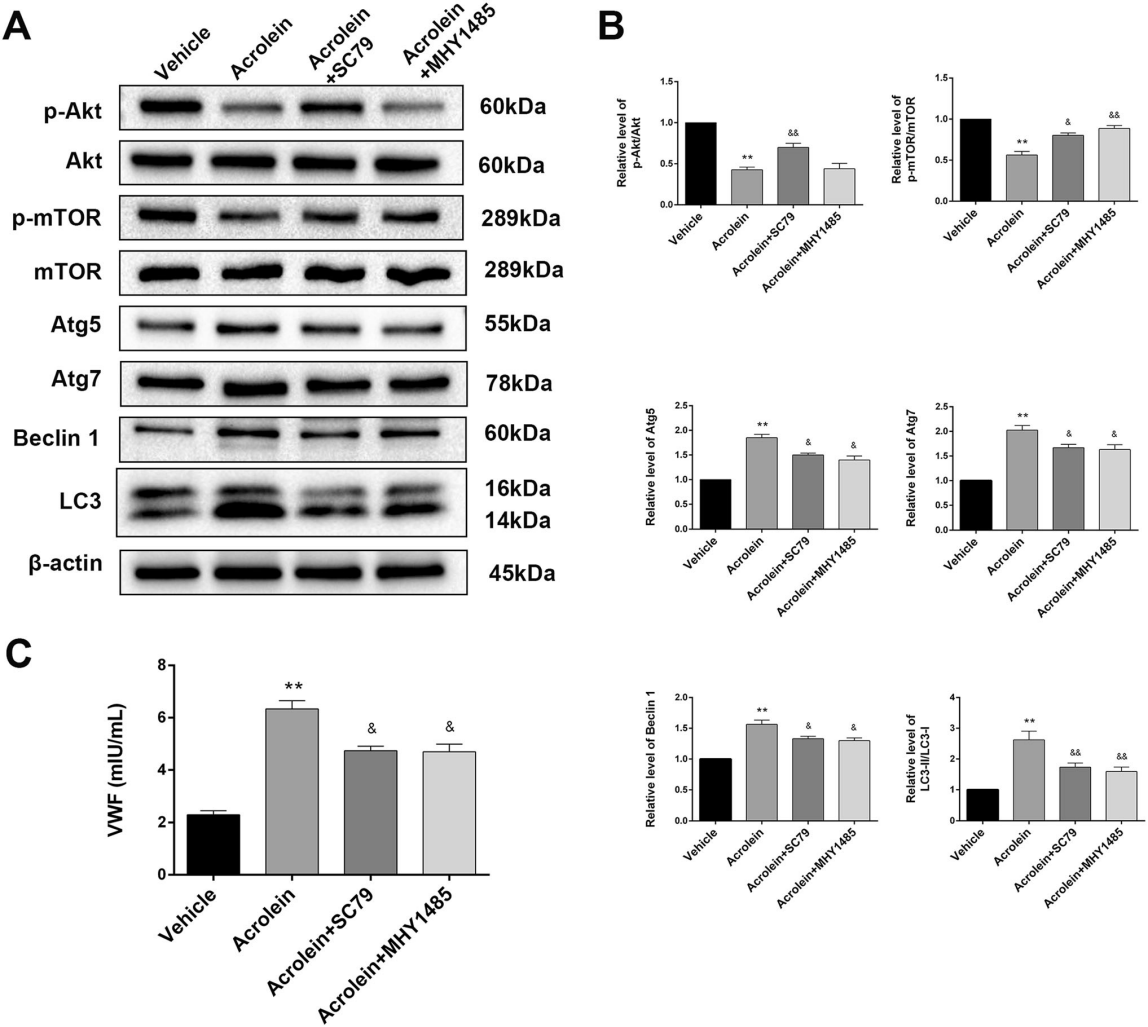
Acrolein activates autophagy *via* the AKT/mTOR pathway.** A** Western blots of p-Akt, Akt, p-mTOR, mTOR, Atg5, Atg7, Beclin 1, and LC3 in each group.** B** Relative levels of these proteins expressed as percentages of β-actin.** C** Effects of SC79 or MHY1485 on acrolein-induced VWF secretion in each group. Values are presented as the mean ± SEM, *n* = 3 per group, *P < 0.05, **P < 0.01 *vs* vehicle group. ^&^*P* < 0.05, ^&&^*P* < 0.01 *vs* acrolein group.

We next explored the possible mechanism by which acrolein activates autophagy. First, GO and KEGG enrichment analyses indicated that the mTOR signaling pathway was one of the most enriched pathways (Fig. 4F). The Akt/mTOR axis is a classic autophagy signaling pathway [38]. Therefore, we hypothesized that acrolein activates autophagy by regulating the Akt/mTOR pathway. After treatment with acrolein (50 μmol/L), the p-Akt/Akt and p-mTOR/mTOR ratios were significantly downregulated (Fig. 6A, B). Then, an Akt activator (SC79) and an mTOR activator (MHY1485) were added after treatment with acrolein. SC79 (5 μg/mL) or MHY1485 (10 μmol/L) abrogated the acrolein-induced reduction in the p-mTOR/mTOR ratio. Accordingly, the expression of Atg5, Atg7, Beclin1, and LC3-II/LC3-I decreased in the SC79+acrolein and MHY1485+acrolein groups compared with the acrolein group (Fig. 6A, B). Furthermore, we measured the level of VWF release in each group. The secretion of VWF was significantly reduced after co-treatment with acrolein and SC79 or MHY1485 (Fig. 6C). These results indicated that the Akt/mTOR-mediated autophagy pathway is involved in the regulation of acrolein-mediated VWF release.

### Acrolein is Upregulated in the Perilesional Cortex, Disrupts the Blood-brain Barrier, and Affects Endothelial Cell Integrity

**Fig. 7:**
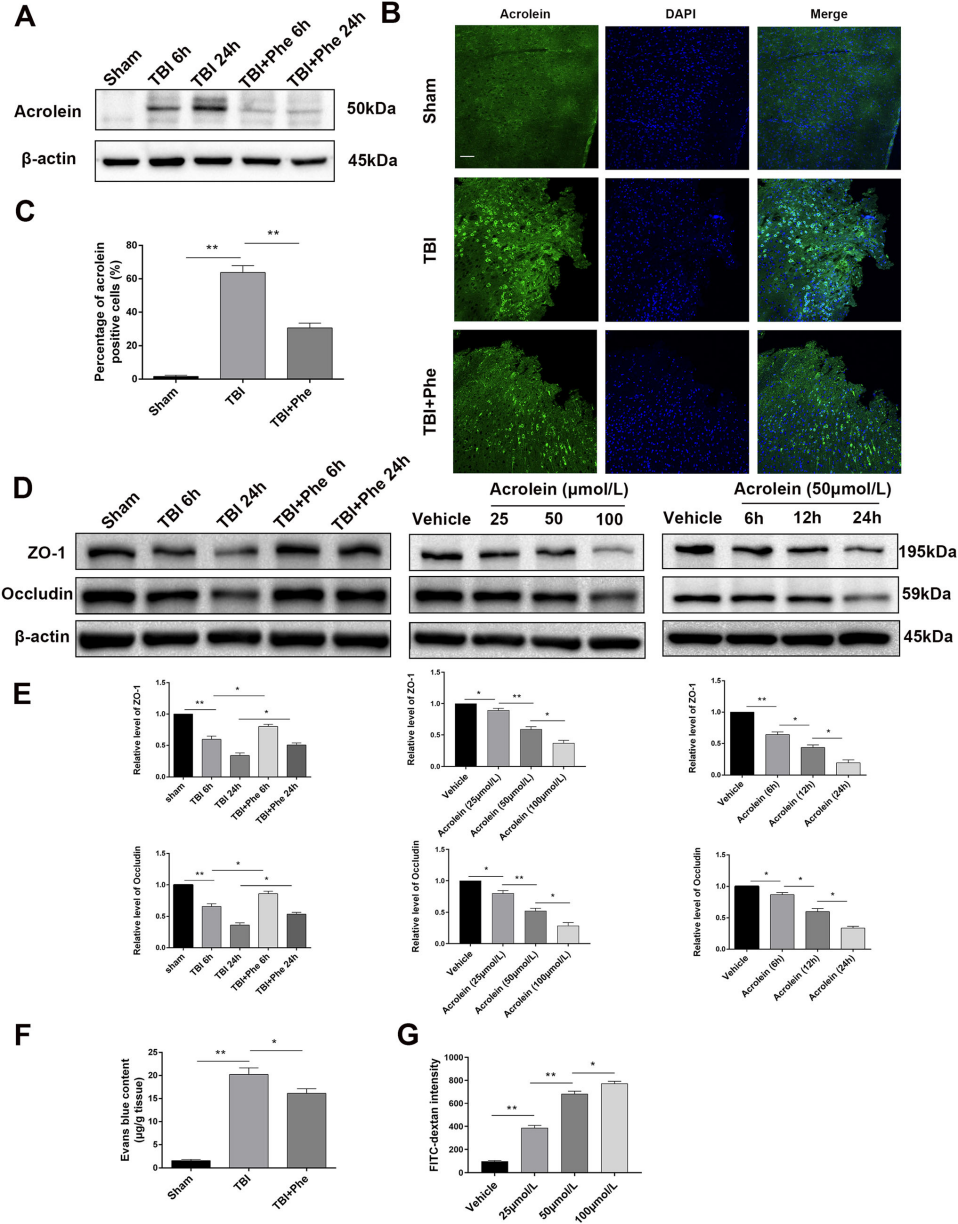
Acrolein is upregulated in the perilesional cortex, disrupts the blood-brain barrier, and affects endothelial cell integrity.** A** Western blots of acrolein in the perilesional cortex of mice at 6 h and 24 h after TBI with or without phenelzine. **B** Representative images of immunofluorescence staining for acrolein (green) in the perilesional cortex 24 h after TBI with or without phenelzine (scale bar, 50 μm). **C** Percentages of acrolein-positive cells. **D, E** Western blots (**D**) and analysis (**E**) of Occludin and ZO-1 expression in each group. **F** Evans blue levels in the brain. **G** Dextran staining intensity in confluent HUVECs in Transwells stimulated with different concentrations of acrolein (25, 50, and 100 μmol/L) and vehicle for 6 h and then incubated with FITC-dextran for 30 min. Values are presented as the mean ± SEM, *n* = 6 per group, *P < 0.05, **P < 0.01.

First, Western blot and immunofluorescence staining were used to assess the alterations in acrolein over time post-TBI in the perilesional cortex of mice with or without phenelzine. The results showed that acrolein was increased in a time-dependent manner in the perilesional cortex, and was cleared by phenelzine (Fig. 7A–C). As described in previous studies, destruction of the BBB plays an important role in TBI secondary coagulopathy [8], so we next explored whether acrolein is involved in the destruction of the BBB. First, we found that acrolein was co-localized with endothelial cells in the cortex (Fig. S2). Then, the administration of phenelzine after TBI abrogated the downregulation of Occludin and ZO-1 (Fig. 7D, E). Next, acrolein treatment of HUVECs reduced the expression of Occludin and ZO-1 in a concentration- and time-dependent manner (Fig. 7D, E). Compared to that of sham mice, TBI significantly increased Evans blue extravasation. Treatment of the mice with phenelzine after TBI significantly mitigated BBB disruption (Figs. 7F and S3). Using a transwell cell migration assay, we found that acrolein contributed to FITC-dextran leakage through the endothelial cell barrier (Fig. 7G). These results revealed that acrolein is significantly upregulated in the perilesional cortex, and contributes to the disruption of the BBB and endothelial cell integrity.

### Phenelzine as a Therapeutic

**Fig. 8:**
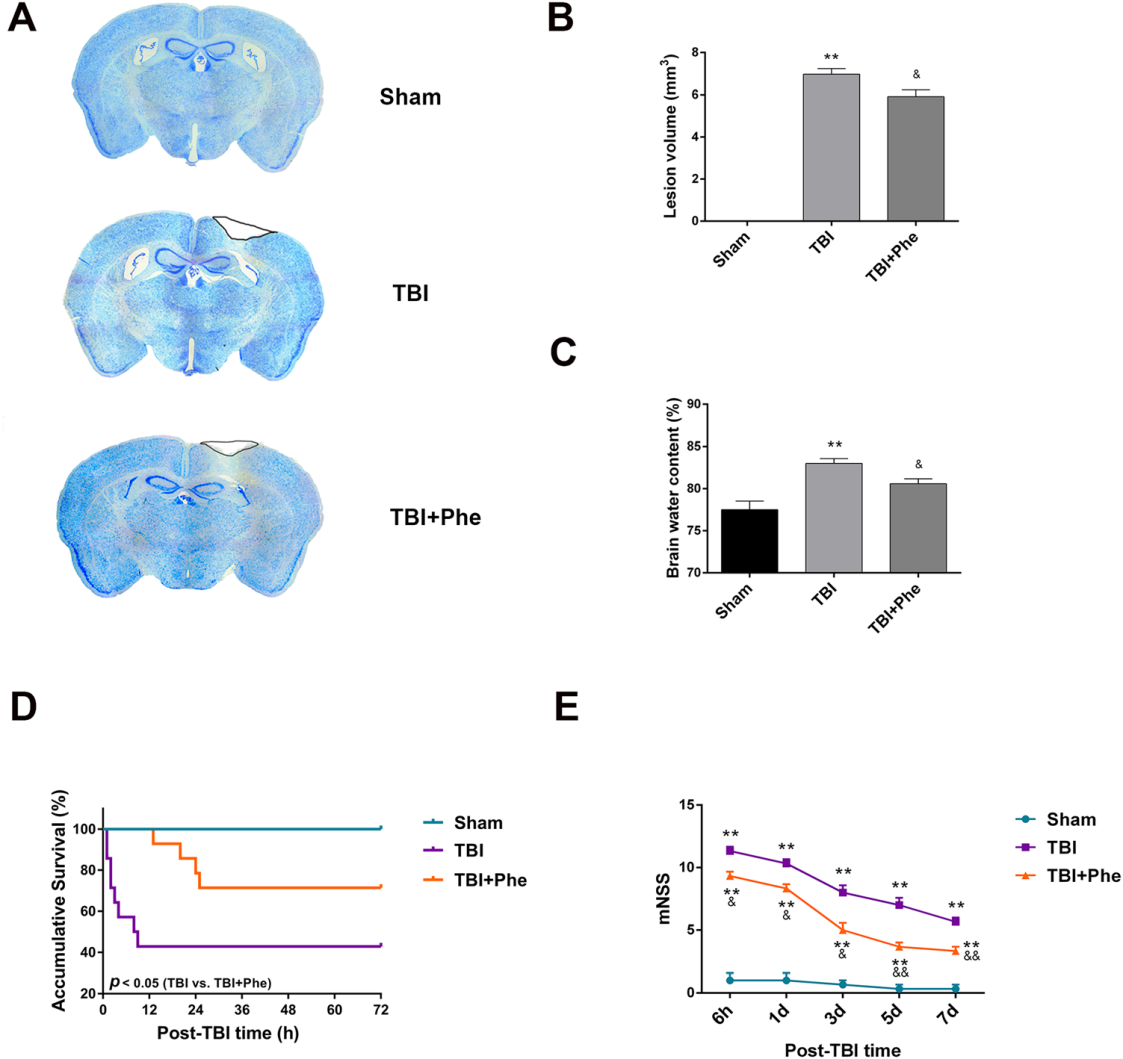
Effects of phenelzine (acrolein scavenger) on lesion volume, brain water content, survival, and neurological function scores in mice after intracranial hemorrhage. **A** Representative images of brains serially sliced and subjected to Nissl staining (*n* = 6 per group). **B** Quantification of lesion volume (*n* = 6 per group). **C** Quantification of brain water content (*n* = 6 per group). **D** Kaplan-Meier survival plots of mice treated with or without phenelzine after TBI and sham mice (*n* = 14 per group). **E** Neurological recovery determined by mNSS at 6 h and 1, 3, 5, and 7 days post-TBI (*n* = 6 per group). Values are presented as the mean ± SEM, *P < 0.05, **P < 0.01 *vs* sham group, ^&^*P* < 0.05, ^&&^*P* < 0.01 *vs* TBI group.

The clearance of acrolein by phenelzine during the acute phase of TBI strongly indicated the therapeutic potential of phenelzine in preventing TBI-induced coagulopathy. We found that a single administration of phenelzine 30 min after TBI reduced circulating acrolein (Fig. 2A) and reversed the trauma-induced hypercoagulable state (Fig. 2B, C). Nissl staining at 24 h after TBI showed that the lesion volume was 15.1% smaller in mice treated with phenelzine than in those treated with vehicle (Fig. 8A, B). Phenelzine also mitigated brain injury and brain edema after TBI (Fig. 8C). By preventing coagulopathy, cerebral edema, and reducing lesion volume, the 7-day mortality was decreased by 28.6% in mice treated with phenelzine, compared to those treated with vehicle (Fig. 8D). Mice treated with phenelzine after TBI had lower neurologic deficit scores than mice in the vehicle-treated group at both time points during the 7-day monitoring period (Fig. 8E). These results indicate the therapeutic and protective effects of phenelzine.

## Discussion

Coagulopathy is a common clinical complication after severe TBI, and is closely associated with intracranial hemorrhage [39] and progressive hemorrhagic injury [40]. Coagulopathy has a detrimental effect on the outcome and overall prognosis of TBI patients [41, 42], leading to an increased risk of mortality and more unfavorable outcomes than in those without coagulopathy [43, 44]. However, the pathophysiological mechanism of TBI-induced coagulopathy remains unclear, resulting in limited measures for prevention and treatment. In an effort to deepen our insight into the molecular mechanisms underlying coagulopathy disease and identify novel therapeutic targets, we investigated the role of lipid-related molecules in TBI. Acrolein, a typical lipid peroxidation product, can lead to thrombosis [20]. Considering its important role in stimulation of the coagulation cascade, we showed for the first time that acrolein is involved in TBI-induced coagulopathy using samples from TBI patients, animal models, and HUVECs.

In the present work, we demonstrated for the first time that acrolein induces systemic coagulopathy after TBI *via* autophagy-dependent secretion of VWF and that acrolein is an interesting potential therapeutic target. Several experimental findings substantiate our conclusions: (1) the plasma acrolein level is increased in TBI patients, and a high plasma acrolein level is associated with the incidence of coagulopathy; (2) acrolein is significantly upregulated in peripheral blood after TBI in mice and contributes to an early hypercoagulable state; (3) acrolein induces coagulopathy by regulating VWF secretion; (4) mRNA-Seq analysis and follow-up experiments demonstrate that acrolein over-activates autophagy by regulating the Akt/mTOR pathway, through which acrolein induces VWF secretion; and (5) acrolein is upregulated in the perilesional cortex, affects endothelial cell integrity, and disrupts the BBB.

To determine the role of acrolein in TBI, we first evaluated its plasma levels in TBI patients and healthy controls, and found that it was higher in TBI patients. We also provided evidence that high plasma acrolein is associated with coagulopathy. Moreover, logistic regression analysis indicated that acrolein is an independent risk factor for coagulopathy. Many studies have explored potential molecules to predict acute traumatic coagulopathy. For example, plasma copeptin levels independently predict acute traumatic coagulopathy and progressive hemorrhagic injury after TBI [45]. Plasma galectin-3 concentrations after TBI are closely related to trauma severity, inflammation, and acute traumatic coagulopathy [46]. The pro-coagulant activity of brain-derived microparticles after TBI has been increasingly studied [24]. It is well established that the brain is rich in lipids [47]; however, the predictive ability and related mechanism of lipid peroxidation products produced after TBI in coagulopathy have not been investigated. The role of acrolein in TBI-induced coagulopathy was indicated in our study. Our clinical evidence supported the notion that high levels of plasma acrolein is an important and powerful predictor of coagulopathy and might play a potential role in the mechanism of coagulopathy. In view of the important role of acrolein in coagulopathy, the clinical importance of acrolein deserves further study.

In a mouse model of TBI, the mice rapidly develop a hypercoagulable state at 6 h, followed by a hypocoagulable state later (at approximately 24 h) [9, 11], consistent with our findings. Our study mainly focused on the time point associated with hypercoagulability, with the goal of preventing and correcting it after TBI. We also evaluated the expression of acrolein in other organs (heart, liver, kidney, intestine, skeletal muscle, and lung) after trauma and found that these organs produced very little (Fig. S4A). We hypothesized that the acrolein produced by the perilesional cortex is an important source of the increased acrolein in peripheral blood. We found that the administration of an acrolein scavenger after TBI improved coagulopathy, and we injected healthy mice with different concentrations of acrolein through the tail vein to simulate elevated acrolein in the circulatory system. Acrolein-induced coagulopathy was concentration-dependent, consistent with our clinical findings.

VWF is a large multimeric glycoprotein that is critical in regulating the balance between bleeding and clotting [48]. It is synthesized only in vascular endothelial cells and megakaryocytes [49]. Although a portion of VWF undergoes basal secretion into the plasma, the majority is stored in the Weibel-Palade bodies (WPBs) in endothelial cells and in the alpha granules of platelets. VWF in the plasma mainly comes from endothelial cells [50]. Its secretion endothelial cells is associated with hypercoagulability and the risk of thrombosis [51]. However, the original initiator of VWF in TBI is unclear. In our study, VWF was increased in the plasma in the acute phase after TBI, and the administration of phenelzine reduced this increase. Similarly, tail vein injection of acrolein caused an increase in VWF in plasma. ADAMTS13 (a disintegrin and metalloproteinase with a thrombospondin type 1 motif, member 13) is mainly synthesized in the liver [52] and is associated with the degradation of thrombogenic VWF multimers [52, 53]. In our study, the administration of rhADAMTS-13 improved the hypercoagulability after tail vein injection of acrolein. Our results suggested that acrolein induces hypercoagulability partly through the secretion of VWF. However, the specific mechanism by which acrolein promotes the secretion of VWF is unclear.

To clarify the specific mechanism by which acrolein promotes VWF release, we determined the transcriptomics changes in HUVECs after acrolein treatment. KEGG analysis indicated that acrolein treatment over-activated autophagy in HUVECs. Interestingly, Takehiro *et al.* demonstrated that WPBs and autophagosomes directly interact and that VWF and WPB remnants are found within autophagosomes. Knockdown or deletion of the essential autophagy genes *Atg5* or *Atg7* impairs the secretion of VWF *in vitro* and *in vivo*, and pharmacological inhibition of autophagic flux leads to a significant prolongation of bleeding time [37]. Proteomic analysis of these secreted endothelial autophagic vacuoles showed that they contain VWF [54]. In our results, we demonstrated that acrolein activated autophagy, and induced the formation of autophagic lysosomes and the release of VWF. The secretion of VWF significantly decreased after acrolein treatment when HUVECs were incubated with autophagy inhibitors. Our results indicated that acrolein regulates the secretion of VWF partly through the autophagy pathway. Furthermore, we explored how acrolein activated autophagy and KEGG analysis showed that the mTOR signaling pathway was one of the most enriched pathways. mTOR plays an important role in regulating autophagy [55], and emerging evidence suggests that Akt is crucial for mTOR phosphorylation and autophagy inhibition [56, 57]. In the present study, treatment with acrolein significantly reduced the phosphorylation of Akt and mTOR. However, co-treatment with acrolein and the AKT activator SC79 or mTOR activator MHY1485 induced a significant increase in the phosphorylation of mTOR. Our studies suggested that acrolein induces autophagy *via* the Akt/mTOR signaling pathway.

We also explored some other possible mechanisms by which acrolein could cause coagulopathy. BBB disruption could contribute to TBI-induced coagulopathy by allowing the release of brain-derived pro-coagulant substances (such as tissue factors, phosphatidylserine, and cardiolipin) into the circulation to induce systemic coagulopathy [8, 9, 24]. Interestingly, in our study the KEGG analysis indicated that acrolein could affect endothelial cell integrity. Here, we demonstrated that acrolein was abundantly produced in the perilesional cortex, disrupted the BBB, and affected endothelial cell integrity. Further disruption of the BBB allowed more acrolein to be released into the circulatory system. In addition, we provided evidence that acrolein is present in blood vessels in the perilesional cortex (Figs S2 and S4B), suggesting that acrolein acts on endothelial cells to some extent. Moreover, acrolein was found in blood vessels in the ipsilateral non-perilesional cortex (Fig. S4C), indicating that acrolein produced at the site of injury can be released into the circulation.

Some limitations should be noted when interpreting the experimental data. First, our study demonstrated that acrolein promotes the secretion of VWF. However, we have not explored whether acrolein affects the synthesis of VWF. Second, our results suggested that acrolein promotes the release of VWF by activating autophagy *via* the AKT/mTOR pathway *in vitro*. In future studies, the use of animals with endothelial cell-specific knockout of autophagy-related genes (such as *Atg5* or *Atg7*) could provide more convincing evidence. Finally, the role of other lipid peroxidation products in TBI-induced coagulopathy deserves further study.

In conclusion, this study showed that acrolein exacerbates systemic coagulopathy in TBI by promoting the secretion of VWF from endothelial cells. At the molecular level, acrolein might regulate VWF release by activating autophagy *via* the AKT/mTOR pathway. Overall, data from our study deserve further investigation to validate acrolein as a promising novel therapeutic target for TBI-induced coagulopathy.

## Supplementary Information

Below is the link to the electronic supplementary material.

Supplementary file1 (PDF 2567 KB)
