# Supplementary material for: Acrolein Induces Systemic Coagulopathy via Autophagy-dependent Secretion of von Willebrand Factor in Mice after Traumatic Brain Injury
Source: Neurosci Bull. 2021 May 3;37(8):1160–75. doi: 10.1007/s12264-021-00681-0 (PMC8353051; doi:10.1007/s12264-021-00681-0)
Supplement: Supplementary file 1 — Supplementary file1 (PDF 2567 KB) [file 12264_2021_681_MOESM1_ESM.pdf]

Supplementary Materials

A

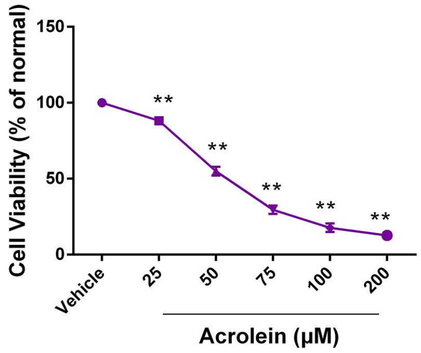

B

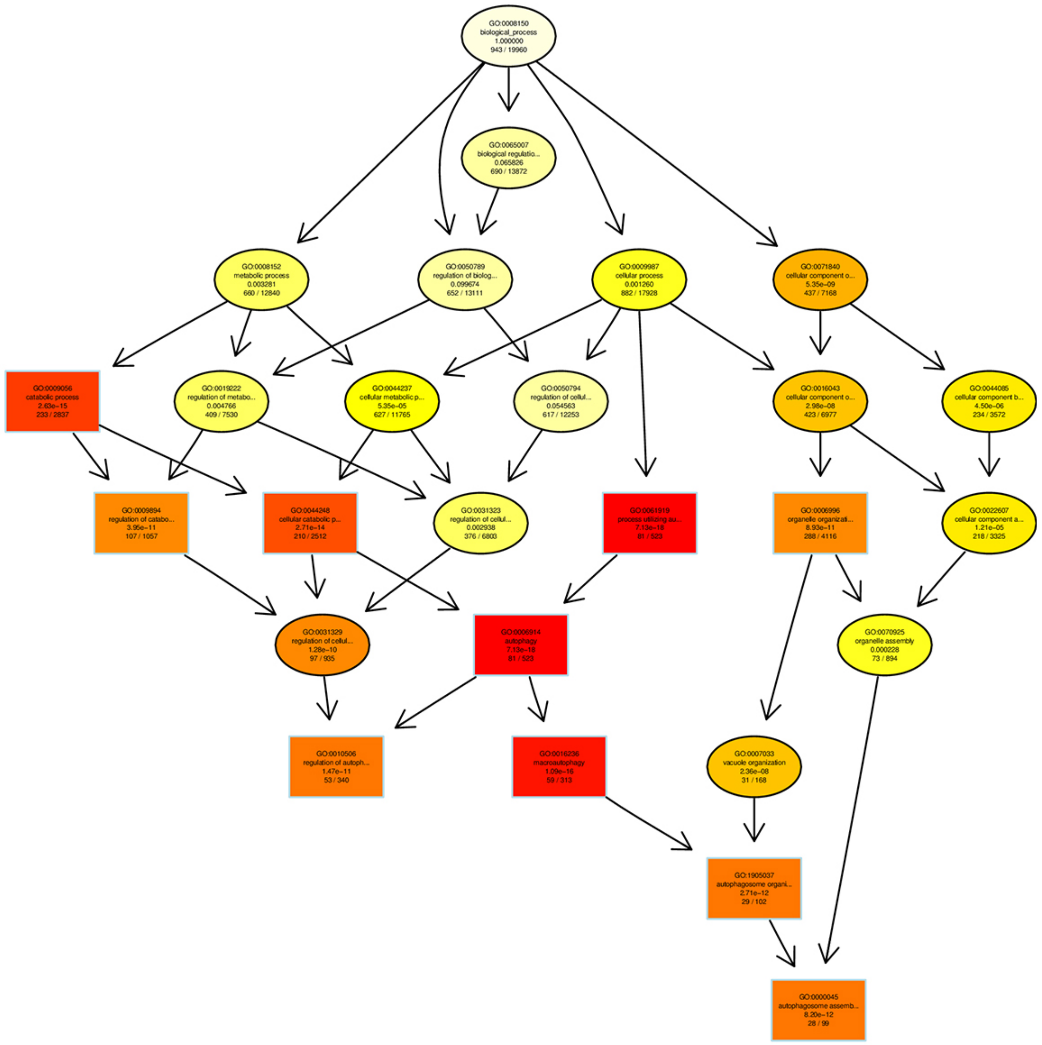

C

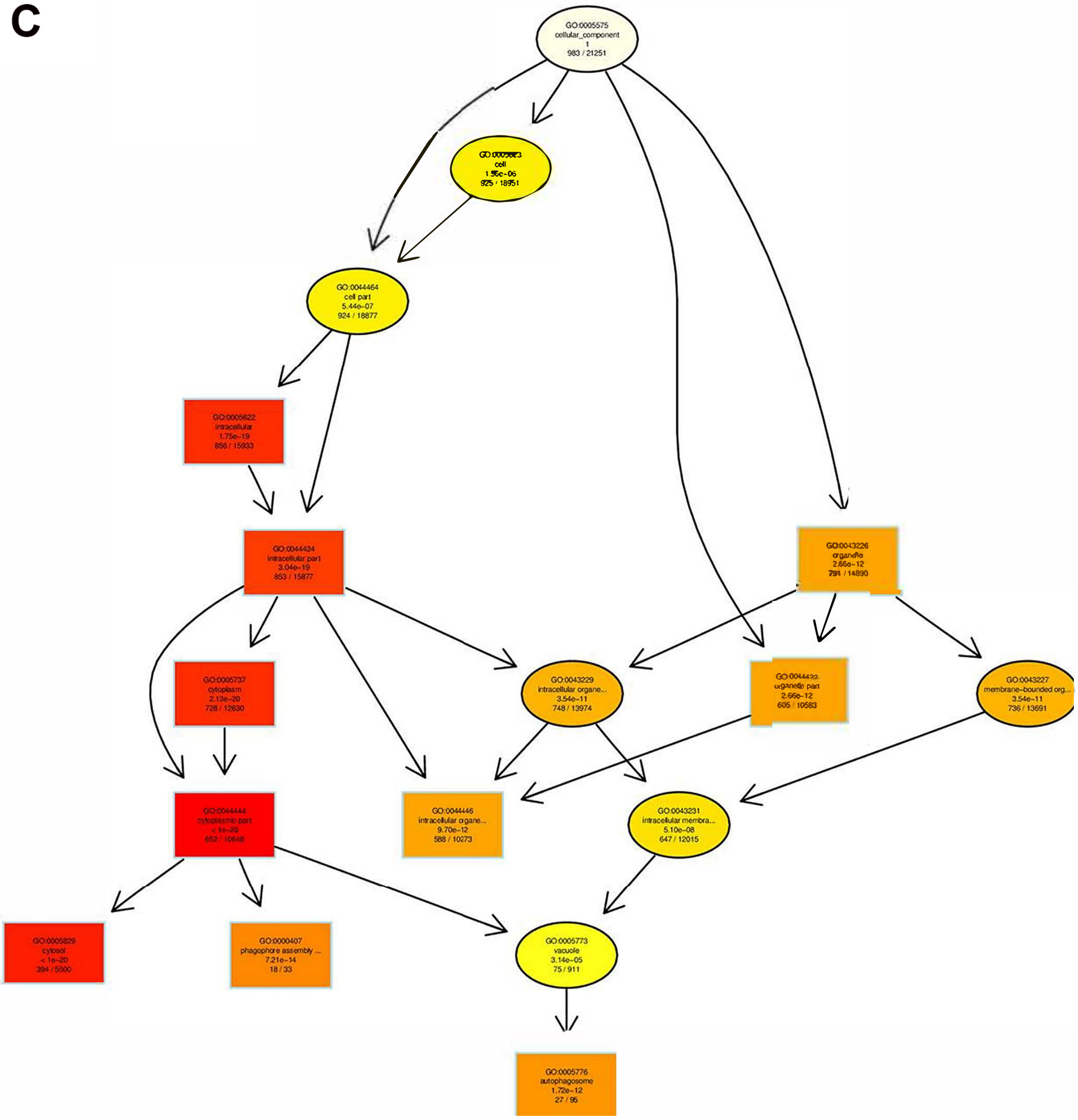
GO:0005575
cellular_component
1
983 / 21251
cell
925 / 18951
GO:0044464
cell part
5.44e-07
924 / 18877
GO:0005622
intracellular
1.75e-19
856 / 15933
GO:0044424
intracellular part
3.04e-19
853 / 15877
GO:0043226
organelle
2.66e-12
791 / 14890
GO:0005737
cytoplasm
2.13e-20
728 / 12630
GO:0043229
intracellular organe...
3.54e-11
748 / 13974
organelle part
2.66e-12
605 / 10583
GO:0043227
membrane-bounded org...
3.54e-11
736 / 13691
GO:0044444
cytoplasmic part
< 1e-20
652 / 10648
GO:0044446
intracellular organe...
9.70e-12
588 / 10273
GO:0043231
intracellular membra...
5.10e-08
647 / 12015
GO:0005829
cytosol
< 1e-20
394 / 5500
GO:0000407
phagophore assembly ...
7.21e-14
18 / 33
GO:0005773
vacuole
3.14e-05
75 / 911
GO:0005776
autophagosome
1.72e-12
27 / 95

D

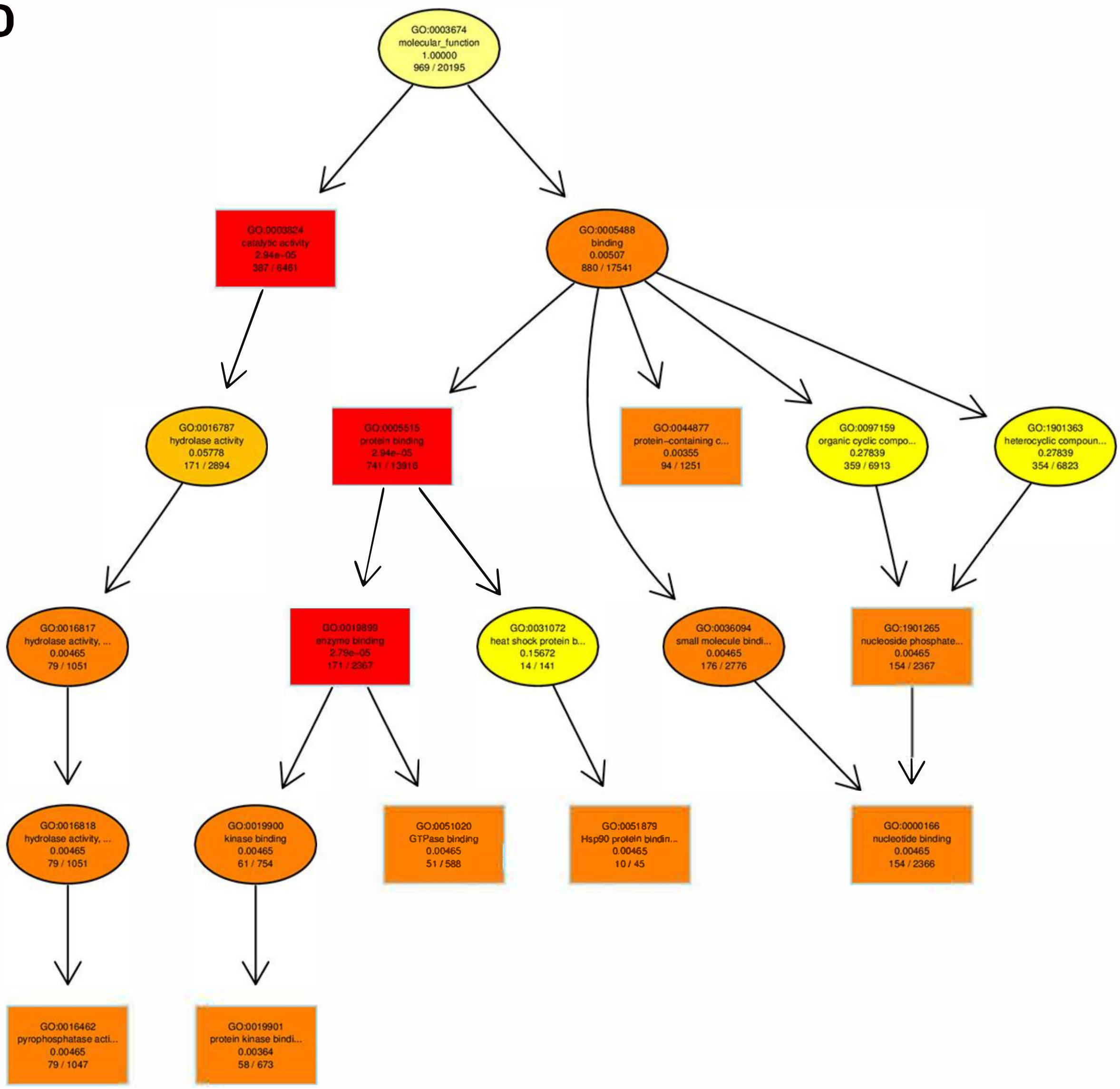

GO:0003674 molecular_function 1.00000 969 / 20195
GO:0003824 catalytic activity 2.94e-05 387 / 6461
GO:0005488 binding 0.00507 880 / 17541
GO:0016787 hydrolase activity 0.05778 171 / 2894
GO:0005515 protein binding 2.94e-05 741 / 13916
GO:0044877 protein-containing c... 0.00355 94 / 1251
GO:0097159 organic cyclic compo... 0.27839 359 / 6913
GO:1901363 heterocyclic compoun... 0.27839 354 / 6823
GO:0016817 hydrolase activity, ... 0.00465 79 / 1051
GO:0019899 enzyme binding 2.79e-05 171 / 2367
GO:0031072 heat shock protein b... 0.15672 14 / 141
GO:0036094 small molecule bindi... 0.00465 176 / 2776
GO:1901265 nucleoside phosphate... 0.00465 154 / 2367
GO:0016818 hydrolase activity, ... 0.00465 79 / 1051
GO:0019900 kinase binding 0.00465 61 / 754
GO:0051020 GTPase binding 0.00465 51 / 588
GO:0051879 Hsp90 protein bindin... 0.00465 10 / 45
GO:0000166 nucleotide binding 0.00465 154 / 2366
GO:0016462 pyrophosphatase acti... 0.00465 79 / 1047
GO:0019901 protein kinase bindi... 0.00364 58 / 673

E

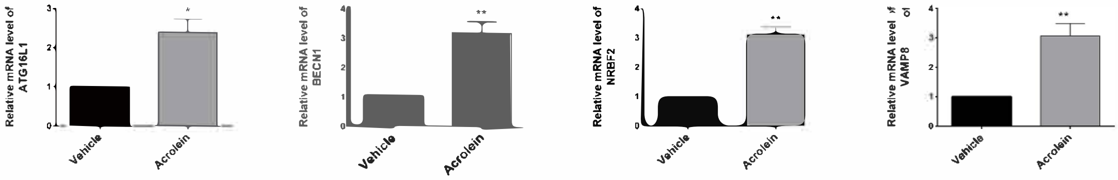

Relative mRNA level of ATG16L1
Relative mRNA level of BECN1
Relative mRNA level of NRBF2
VAMP8
*
**
**
**
Vehicle
Acrolein

Fig S1 mRNA-Seq analysis showing that acrolein activates autophagy. **A** CCK8 assays of cell viability in HUVECs treated with acrolein (25, 50, 75, 100, and 200 μmol/L) and vehicle for 6 h. **B–D** GO and pathway analysis of biological process (**B**), cellular components (**C**), and molecular function (**D**). **E** qPCR validates the changes in autophagy-related gene expression. Values are represented as the mean ± SEM, *P <0.05 and **P <0.01 *vs* vehicle group.

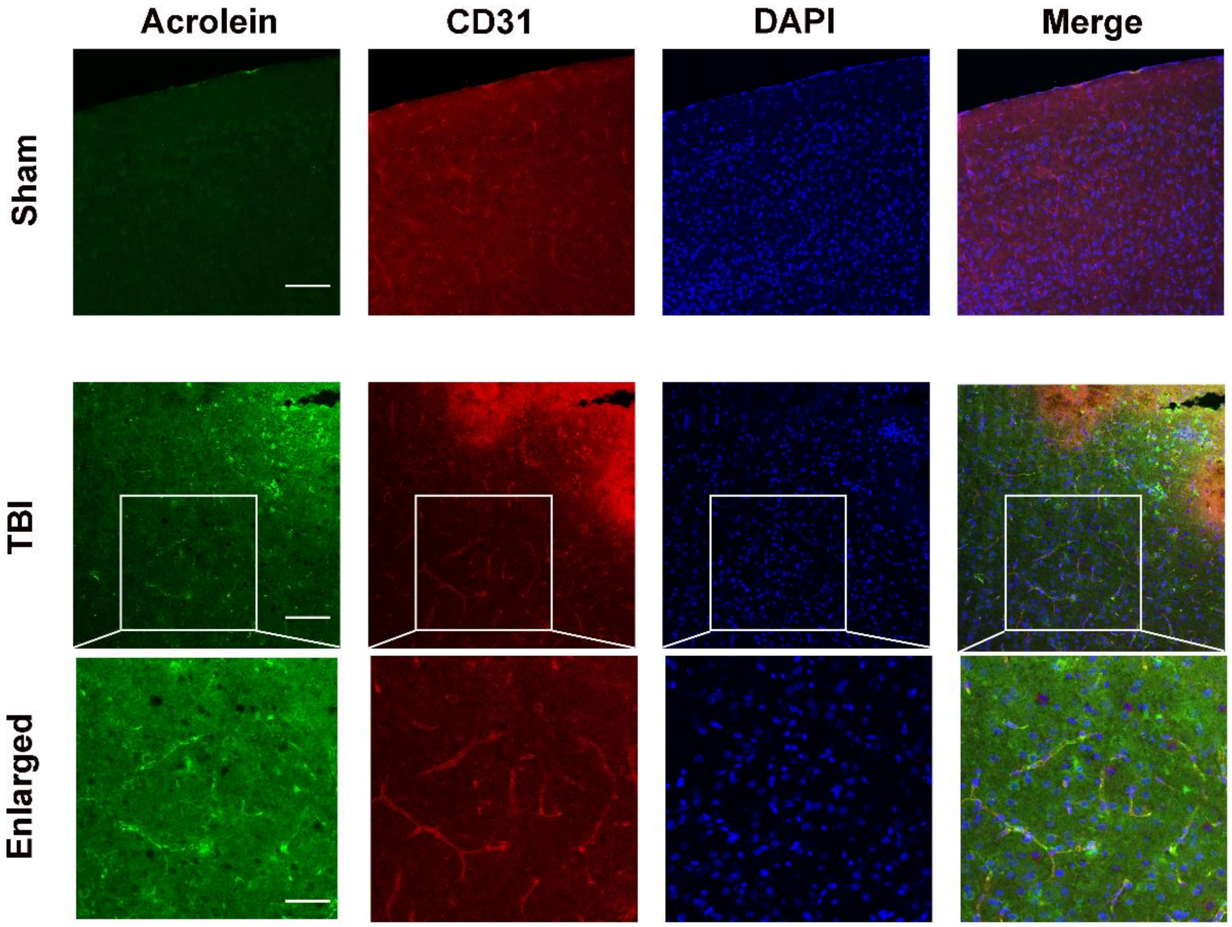

Fig. S2 Representative images of immunofluorescence staining for acrolein (green) and CD31 (red) in the perilesional cortex 24 h after TBI (scale bars, 100 μm; enlarged, 50 μm).

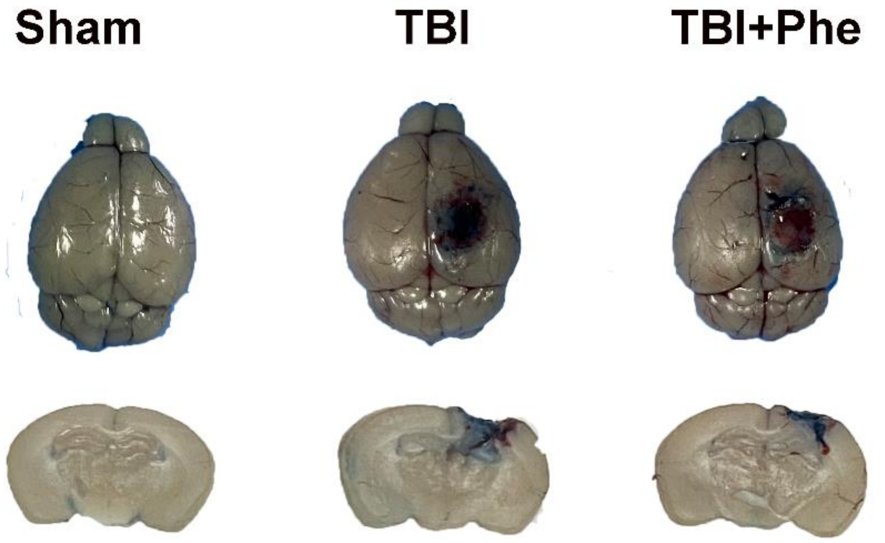

Fig. S3 Representative images of the dorsal surface and coronal section showing Evans blue extravasation.

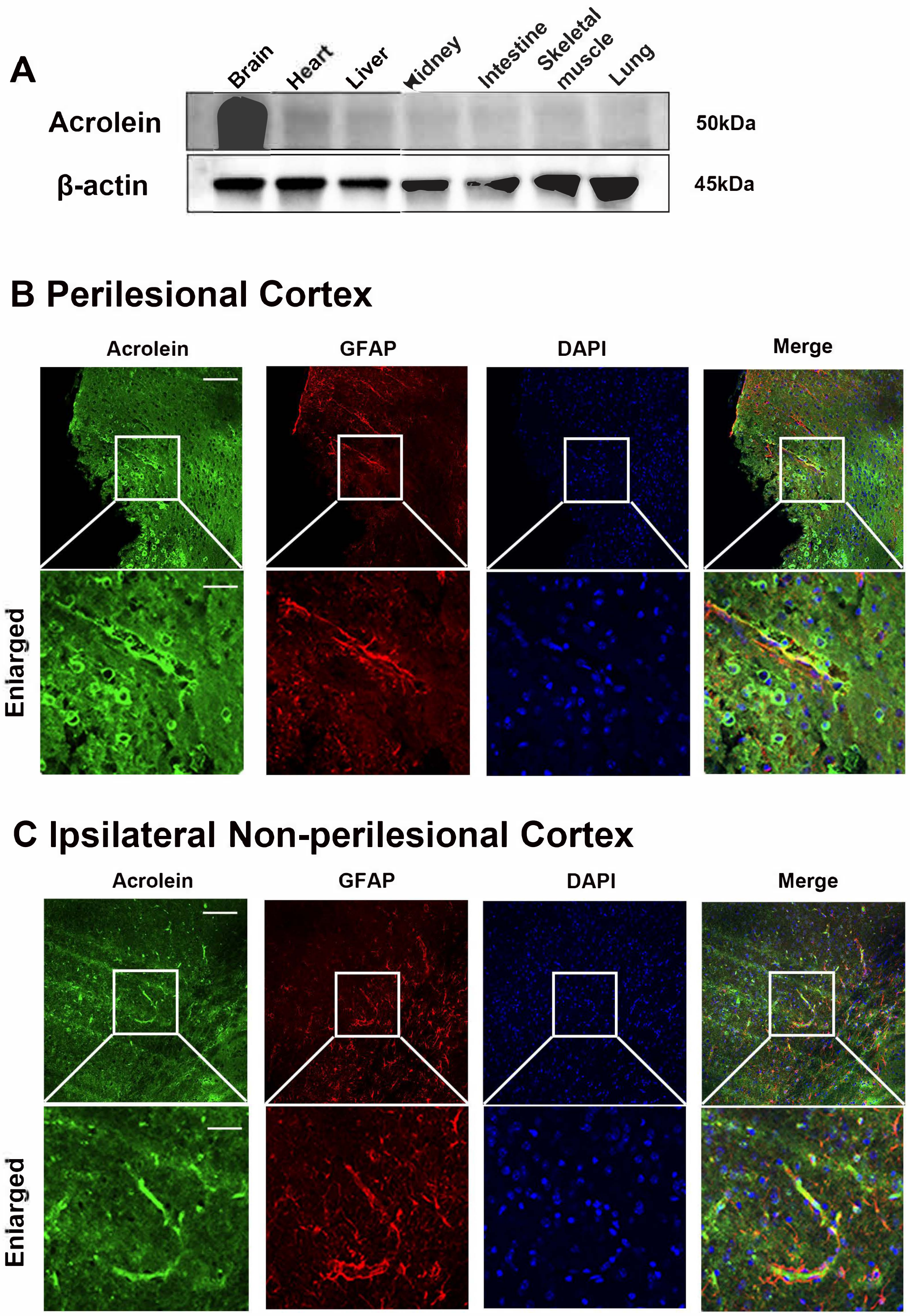
A
Brain
Heart
Liver
Kidney
Intestine
Skeletal muscle
Lung
Acrolein
β-actin
50kDa
45kDa
B Perilesional Cortex
Acrolein
GFAP
DAPI
Merge
Enlarged
C Ipsilateral Non-perilesional Cortex
Acrolein
GFAP
DAPI
Merge
Enlarged

Fig. S4 A Western blots of acrolein in the brain, heart, liver, kidney, intestine, skeletal muscle, and lung after TBI. **B, C** Representative images of double immunofluorescence staining for acrolein (green) and GFAP (red) in perilesional cortex (**B**) and ipsilateral non-perilesional cortex (**C**) (scale bars, 100 μm; enlarged, 30 μm).

Table S1 Sequences of primers used in qRT-PCR

Name	Forward primer (5′–3′)	Reverse primer (5′–3′)
Beclin-1	GGGCTCCCGAGGGATGG	GCTGTTGGCACTTTCTGTGG
ATG16L1	TGGGGAGTTAGCTCAACTGGTG	AAGAGACAGAGCGTCTCCCA
Vamp8	GGAAGCCACATCTGAGCACT	AGCCCACTCTAAGGACCCAA
NRBF2	AAGGCTGCAGCATATCTTTCTGA	TCTCAGGCAGGCATTTCTCTG
